# Hereditary Myopathy With Early Respiratory Failure Associated With an Incidental *COL4A5* Variant: A Case Report

**DOI:** 10.1155/crig/1630468

**Published:** 2026-02-10

**Authors:** Ursula Abu Nahla, Rahaf Bleibel, Mai Arafeh, Saif Khaled Abdalhadi Azzam, Lina Barhoum, Mostafa Ibraheem, Motaz Altamimi, Bashar Sultan, Orwa Al Fallah

**Affiliations:** ^1^ Department of Pediatrics, Hebron University, Hebron, West Bank, State of Palestine, hebron.edu; ^2^ Department of Clinical Medical Sciences, Faculty of Medicine and Health Sciences, Palestine Polytechnic University, Hebron, P720, West Bank, State of Palestine, ppu.edu; ^3^ Department of Pediatric Neurology, Hebron University, Hebron, West Bank, State of Palestine, hebron.edu; ^4^ Radiology Department, Al-Ahli Hospital, Hebron, 9020000, West Bank, State of Palestine, ahlihospital.com

**Keywords:** *COL4A5*, HMERF, incidental variant, muscle weakness, respiratory failure, *TTN*, VUS

## Abstract

**Background:**

Hereditary myopathy with early respiratory failure (HMERF) is a rare autosomal dominant disorder caused by *TTN* variants. *COL4A5* mutations are linked to X‐linked Alport syndrome.

**Case Presentation:**

A 34‐year‐old male developed progressive lower limb weakness, gait disturbance, nocturnal hypoventilation, and calf hypertrophy. Family history revealed similar symptoms in his mother and sister. Examination showed absent reflexes; MRI demonstrated muscle atrophy and fatty replacement; needle electromyography (EMG) was performed and showed findings consistent with advanced myopathy; however, it was not used as a primary diagnostic tool. Whole‐exome sequencing identified a pathogenic *TTN* variant (c.95126C > G, p.Pro31709Arg), confirming HMERF. A hemizygous *COL4A5* variant (c.4891C > T, p.Arg1631Cys) was also detected but lacked clinical correlation.

**Discussion and Conclusion:**

This case illustrates a classic HMERF phenotype confirmed genetically, with an incidental *COL4A5* variant of uncertain significance. It underscores the importance of genomic testing in atypical neuromuscular presentations and the need for cautious interpretation of incidental findings.

## 1. Introduction

Hereditary myopathy with early respiratory failure (HMERF) is a rare, progressive muscle disorder caused by mutations in the *TTN* gene, which encodes titin, a critical protein for muscle elasticity and function [1]. Typically manifesting after the age of 30, HMERF is inherited in an autosomal dominant fashion and is characterized by progressive weakness that initially affects the distal leg and respiratory muscles, eventually leading to generalized weakness and respiratory insufficiency [2]. Variants in *COL4A5* are associated with X‐linked Alport syndrome (AS), a condition that primarily affects renal, auditory, and ocular systems [3]. While AS generally presents with progressive renal dysfunction, sensorineural hearing loss, and ocular abnormalities, its clinical expressivity is highly variable. In the present case, a pathogenic *TTN* variant confirmed the diagnosis of HMERF, and an additional *COL4A5* variant of uncertain or likely benign significance was identified [4]. This highlights the importance of comprehensive genomic testing in neuromuscular disorders while underscoring the need for cautious interpretation of incidental genetic findings that lack clear clinical correlation [5].

## 2. Case Presentation

A 34‐year‐old male with a normal perinatal and developmental history presented with progressive weakness of the lower limbs, which began at age 30. Initially, he experienced gait abnormalities, difficulty climbing stairs, and mild challenges with transitioning between sitting and standing positions. Over the past year, the weakness worsened, rendering him unable to change positions independently. He reported a weight loss of 16 kg since symptom onset and sleep‐disordered breathing consistent with nocturnal hypoventilation. Notably, there was no involvement of the upper limbs.

The family history was significant: his mother had died from an undiagnosed progressive neuromuscular disorder leading to respiratory failure, while his older sister exhibited similar symptoms but had not sought medical attention. There was no known parental consanguinity.

On physical examination, the patient exhibited bilateral calf hypertrophy, limited dorsiflexion, and absent deep tendon reflexes throughout the lower limbs. There were no clinical signs of axial muscle weakness. Neck flexion and extension were preserved. He was able to rise from a supine position independently without upper limb support, and no Gowers’ sign was observed.

**FIGURE 1:**
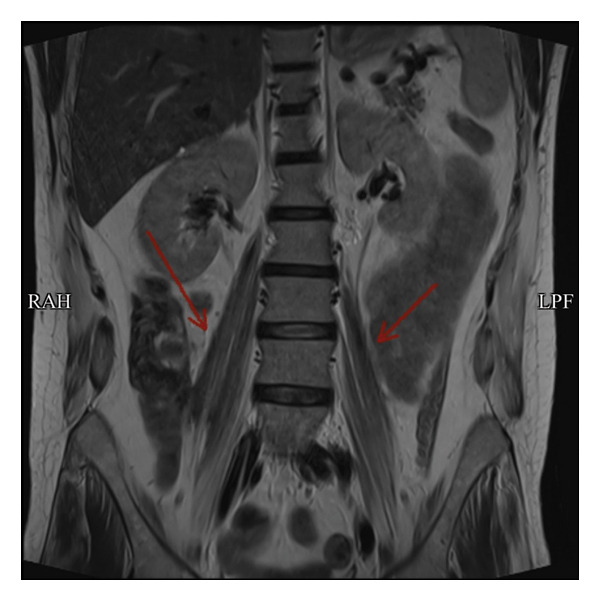
Coronal T2‐weighted MRI image showing diffuse atrophy and fatty replacement of the psoas muscles (indicated by the red arrow).

**FIGURE 2:**
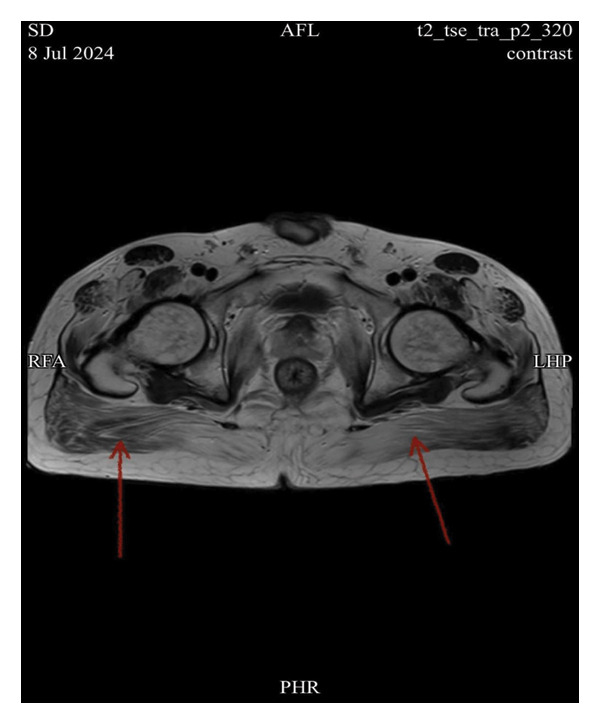
Axial T1‐weighted MRI image demonstrating diffuse atrophy and fatty replacement of the gluteal muscles (indicated by the red arrow).

Imaging studies included magnetic resonance imaging (MRI) of the lumbar spine using axial and sagittal T1‐ and T2‐weighted sequences, sagittal T2 fat‐saturated, and coronal T2 sequences. MRI revealed diffuse atrophy and fatty replacement of the psoas and gluteal muscles, findings consistent with advanced myopathic changes (Figures 1 and 2). Multiple disc herniations were identified at several levels, including D11–D12 and L2–L3; however, none caused significant central canal or neural foraminal stenosis. A few bilateral renal cysts were noted as incidental findings.

Nerve conduction studies were largely within normal limits, except for reduced compound muscle action potential (CMAP) amplitudes in the bilateral fibular motor nerves (left: 1.47 mV; right: 1.29 mV), interpreted as reflecting axonal loss or severe muscle involvement rather than demyelination [6]. Needle electromyography (EMG) of distal muscles revealed the absence of motor unit potentials in the tibialis anterior, consistent with advanced chronic myopathic changes, while the tibialis posterior and abductor digiti minimi showed normal motor unit potentials.

Whole‐exome sequencing (WES) identified a heterozygous pathogenic variant in the *TTN* gene (c.95126C > G, p.Pro31709Arg), confirming the diagnosis of HMERF (1,3). In addition, a hemizygous *COL4A5* variant (c.4891C > T, p.Arg1631Cys) was detected. This variant has conflicting interpretations in ClinVar [7], is classified as likely benign in VarSome, and has been reported in multiple hemizygous males in gnomAD. In the absence of renal, auditory, or ocular abnormalities, the variant was interpreted as incidental and of uncertain or likely benign significance [8, 9].

Laboratory evaluation revealed preserved renal function (blood urea nitrogen 13.4 mg/dL, serum creatinine 0.52 mg/dL, and normal eGFR), no hematuria or proteinuria, and normal blood pressure. Audiological and ophthalmological assessments were not performed, but the patient reported no hearing impairment or visual complaints. There were no clinical features suggestive of retinopathy, lens abnormalities, or corneal erosions.

Taken together, the clinical findings, imaging results, and genetic testing confirmed a diagnosis of HMERF. The additional *COL4A5* variant was best regarded as incidental. This case underscores the importance of correlating genetic results with clinical observations and highlights the need for cautious interpretation of variants lacking clear phenotypic expression in complex neuromuscular disorders [5].

### 2.1. Genetic Analysis

The genetic analysis revealed variants in both COL4A5 and TTN. To our knowledge, this is the first reported case worldwide documenting the co‐occurrence of these variants in the same patient. Genomic DNA was isolated from whole blood samples using standard procedures. WES was performed using the ExoSeq COMP method, which utilizes next‐generation sequencing (NGS) technology to analyze the exome of the proband.

In addition, NGS‐based copy number variation (CNV) analysis was conducted to identify potential structural variations. This approach enabled the detection of the mutation.

WES revealed a missense variant in the TTN gene, associated with HMERF. The variant is located at Chr2:179410837 and is annotated as c.95126C > G (p. Pro31709Arg) in the NM_001267550 transcript (Accession: VCV000132132.12, dbSNP: rs869320739) [6]. The inheritance pattern follows an autosomal dominant mode, and the individual was found to be heterozygous for the variant, so there is a 50% chance that any sibling will have the same variant, and there is a 50% chance that the variant can be passed on to the children of the patient. Based on classification databases (ClinVar, VarSome, and Franklin), the variant is considered likely pathogenic.

Incidentally, AS was discovered during extended genetic analysis, which revealed a variant in the COL4A5 gene. This variant is located at ChrX:107938566 and is annotated as c.4891C > T (p. Arg1631Cys) in the NM_033380.3 transcript (Accession: VCV000994529.4, dbSNP: rs865842167). The inheritance pattern follows an X‐linked dominant mode, and the individual was found to be hemizygous for the variant, so none of the sons of the patient will get the variant, but all the daughters of the patient will get the same variant. Based on classification databases (ClinVar, VarSome, and Franklin), the variant is considered likely pathogenic.

All the genetic variants identified are summarized in Supporting Table 1, which provides details on the gene, transcript, variant, coverage, zygosity, inheritance pattern, associated disease, classification, database references, and accession numbers.

## 3. Discussion

HMERF is a distinct titinopathy within the spectrum of myofibrillar myopathies. It is typically characterized by progressive weakness of the distal lower limbs, calf hypertrophy, and early involvement of respiratory muscles, often manifesting in the third to fifth decades of life [1]. The underlying pathogenic mechanism involves mutations in the *TTN* gene, which encodes titin, a giant sarcomeric protein essential for muscle elasticity, structural integrity, and mechanosensing. Pathogenic variants disrupt titin’s function, leading to sarcomeric disorganization, myofibrillar degeneration, and progressive muscle weakness [1].

EMG findings in HMERF often demonstrate chronic myopathic changes, including reduced motor unit recruitment and fibrotic replacement of muscle tissue. In our patient, the absence of motor unit potentials in the tibialis anterior muscle provided strong electrophysiological evidence of advanced myopathy [6].ِAlthough needle EMG was performed; however, we did not rely on it for diagnostic conclusions, as it is not suitable for detecting fibrotic changes or assessing spontaneous activity in fibrotic muscles. These findings were corroborated by MRI findings of diffuse fatty replacement and atrophy, which are consistent with the imaging phenotype described in previous cohorts [1]. Genetic confirmation was achieved by identifying the pathogenic *TTN* variant c.95126C > G (p.Pro31709Arg), a mutation previously reported in association with HMERF [3].

In addition to the *TTN* variant, a hemizygous *COL4A5* variant (c.4891C > T, p.Arg1631Cys) was detected. Variants in *COL4A5* are classically associated with X‐linked Alport syndrome, which manifests with progressive renal dysfunction, sensorineural hearing loss, and ocular abnormalities [3, 4]. However, the present variant has conflicting interpretations in ClinVar, is classified as likely benign in VarSome, and has been observed in multiple hemizygous males in gnomAD without clear pathogenic correlation [7]. Savige et al. highlighted that pathogenic *COL4A5* variants most frequently involve glycine substitutions within the Gly–X–Y motif of the collagen triple helix, whereas the current variant lies in the NC1 domain, further supporting its classification as a variant of uncertain significance [8]. Thus, in the absence of renal, auditory, or ocular manifestations, this finding is best regarded as incidental rather than diagnostic of AS [9].

This case underscores the importance of comprehensive genomic testing in patients with atypical neuromuscular presentations [5]. WES not only confirmed the diagnosis of HMERF but also revealed an incidental variant, highlighting the need for cautious interpretation of genetic findings that lack clear clinical correlation. Overinterpretation of incidental variants may lead to unnecessary anxiety, misdiagnosis, or inappropriate management, emphasizing the role of multidisciplinary review and variant classification frameworks such as ACMG/AMP guidelines [8].

From a clinical management perspective, patients with HMERF require regular respiratory monitoring to detect hypoventilation early, as respiratory failure is a major cause of morbidity and mortality [10]. Noninvasive ventilation should be initiated promptly when nocturnal hypoventilation or daytime hypercapnia is detected. Supportive interventions, including physiotherapy, targeted exercise programs to strengthen ankle and tibial muscles, and bracing with ankle–foot orthoses, can help maintain ambulation and delay functional decline [10, 11]. In selected cases, surgical interventions such as posterior tibial tendon transfer may be considered to correct foot drop and improve gait mechanics [11].

Taken together, this case illustrates a well‐documented phenotype of HMERF confirmed by genetic testing, with an incidental *COL4A5* variant of uncertain significance. It highlights the dual importance of advanced genomic diagnostics and careful clinical correlation, ensuring that incidental findings are interpreted within the broader clinical context. Ultimately, multidisciplinary care including neurologists, geneticists, physiotherapists, and respiratory specialists remains essential to optimize outcomes in rare neuromuscular disorders (see Table 1).

**TABLE 1:** Detected genetic variants in the patient, classified according to ACMG criteria and confirmed in ClinVar, VarSome, Franklin, and dbSNP databases.

Variant details		
Gene ID	*TTN*	*COL4A5*
Transcript	NM_001267550.2	NM_033380.3
Location	Chr2:179410837	ChrX:107938566
HGVSc and HGVSp	c.95126C > G p.Pro31709Arg	c.4891C > T p.Arg1631Cys
Coverage	26:26	0:58
Zygosity	Heterozygous	Hemizygous
Inheritance pattern	Autosomal dominant	X‐Linked dominant
Disease	Tibial muscular dystrophy, tardive; myopathy, myofibrillar, 9, with early respiratory failure	Alport syndrome 1, X‐linked
Classification	Likely pathogenic (PS4, PP5, PS3, PM2, and PP3)	Likely pathogenic (PP3, PM2, PP2, PM1, and PP5)
Accession	VCV000132132.12	VCV000994529.4
dbSNP	rs869320739	rs865842167
Classification database	ClinVar, VarSome, and Franklin	ClinVar, VarSome, and Franklin

*Note:* Accession numbers are provided for each variant.

## 4. Conclusion

This report presents a well‐documented case of HMERF, confirmed by the identification of a pathogenic *TTN* variant. An additional *COL4A5* variant was detected; however, in the absence of renal, auditory, or ocular manifestations, this finding is best regarded as incidental and of uncertain or likely benign significance. The case underscores the critical role of genomic diagnostics in patients with atypical neuromuscular presentations and highlights the importance of cautious interpretation of genetic variants that lack clear clinical correlation. Multidisciplinary care and personalized management remain essential to optimize outcomes in rare neuromuscular disorders.

## Author Contributions

Ursula Abu Nahla contributed to conceptualization, data collection, visualization, writing the original draft, literature review, initial analysis of clinical findings and manuscript editing andformatting. Rahaf Bleibel was involved in writing the original draft and initial analysis of clinical findings. Mai Arafeh was involved in writing the original draft and initial analysis of clinical findings. Saif Khaled Abdalhadi Azzam was involved in writing the original draft and manuscript formatting. Lina Barhoum contributed to writing the original draft. Mostafa Ibraheem participated in writing the original draft. Motaz Altamimi provided supervision and contributed to the review of the final draft. Bashar Sultan provided supervision for the clinical aspects of the report. Orwa Al Fallah provided radiological expertise and image interpretation.

## Funding

No specific grant from funding agencies was received for this work.

## Disclosure

All authors have read and approved the final manuscript and agree to take full responsibility for all aspects of the research to ensure its accuracy and integrity.

## Ethics Statement

Our institution does not require ethical approval for reporting individual cases or case series.

## Consent

Written informed consent was obtained from the patient for their anonymized information to be published in this article.

## Conflicts of Interest

The authors declare no conflicts of interest.

## Supporting Information

Supporting Table 1: Detailed list of all genetic variants identified by WES in the patient.

## Supporting information


**Supporting Information** Additional supporting information can be found online in the Supporting Information section.

## Data Availability

All genetic variants described in this case report are summarized in Supporting Table 1. These variants have been previously reported and are publicly available in ClinVar, VarSome, Franklin, and dbSNP databases, with accession numbers provided in the table (*TTN*: VCV000132132.12, rs869320739; *COL4A5*: VCV000994529.4, rs865842167). No novel DNA, RNA, or protein sequences were generated in this study. The patient’s raw sequencing data are not publicly available due to privacy and ethical restrictions.
