# Supplementary material for: Hereditary Myopathy With Early Respiratory Failure Associated With an Incidental COL4A5 Variant: A Case Report
Source: Case Rep Genet. 2026 Feb 10;2026:1630468. doi: 10.1155/crig/1630468 (PMC12890873; doi:10.1155/crig/1630468)
Supplement: Supplementary file 1 — Supporting Information Additional supporting information can be found online in the Supporting Information section. [file CRIG-2026-1630468-s001.docx]

| **Variant Details** | | |
| --- | --- | --- |
| **Gene ID** | TTN | COL4A5 |
| **Transcript** | NM_001267550.2 | NM_033380.3 |
| **Location** | Chr2:179410837 | ChrX:107938566 |
| **HGVSc and HGVSp** | c.95126C>G  p.Pro31709Arg | c.4891C>T  p.Arg1631Cys |
| **Coverage** | 26:26 | 0:58 |
| **Zygosity** | Heterozygous | Hemizygous |
| **Inheritance Pattern** | Autosomal Dominant | X-Linked Dominant |
| **Disease** | Tibial muscular dystrophy, tardive; Myopathy, myofibrillar, 9, with early respiratory failure | Alport Syndrome 1, X-Linked |
| **Classification** | Likely Pathogenic (PS4, PP5, PS3, PM2 and PP3) | Likely Pathogenic (PP3, PM2, PP2, PM1 and PP5) |
| **Accession** | VCV000132132.12 | VCV000994529.4 |
| **dbSNP** | rs869320739 | rs865842167 |
| **Classification Database** | ClinVar, Varsome and Franklin | ClinVar, Varsome and Franklin |

**Table 1.** *Detected genetic variants in the patient, classified according to ACMG criteria and confirmed in ClinVar, VarSome, Franklin, and dbSNP databases. Accession numbers are provided for each variant.*
